# MHC-Based Mate Choice in Wild Golden Snub-Nosed Monkeys

**DOI:** 10.3389/fgene.2020.609414

**Published:** 2020-12-21

**Authors:** Bing-yi Zhang, Han-yu Hu, Chun-mei Song, Kang Huang, Derek W. Dunn, Xi Yang, Xiao-wei Wang, Hai-tao Zhao, Cheng-liang Wang, Pei Zhang, Bao-guo Li

**Affiliations:** ^1^Shaanxi Key Laboratory for Animal Conservation, College of Life Sciences, Northwest University, Xi’an, China; ^2^Shaanxi Institute of Zoology, Xi’an, China; ^3^Xi’an Branch of Chinese Academy of Sciences, Xi’an, China

**Keywords:** mate choice, *Rhinopithecus roxellana*, major histocompatibility complex, extra-pair paternity, intermediate dissimilarity

## Abstract

The genes of the major histocompatibility complex (MHC) are an important component of the vertebrate immune system and play a significant role in mate choice in many species. However, it remains unclear whether female mate choice in non-human primates is based on specific functional genes and/or genome-wide genes. The golden snub-nosed monkey (*Rhinopithecus roxellana*) lives in a multilevel society, which consists of several polygynous one-male-several-female units. Although adult females tend to mainly socialize with one adult male, females often initiate extra-pair copulations with other males resulting in a high proportion of offspring being fathered by extra-pair males. We investigated the effects of adaptive MHC genes and neutral microsatellites on female mate choice in a wild *R. roxellana* population. We sequenced 54 parent-offspring triads using two MHC class II loci (*Rhro-DQA1* and *Rhro-DQB1*) and 20 microsatellites from 3 years of data. We found that the paternities of offspring were non-randomly associated with male MHC compositions not microsatellite genotypes. Our study showed that the fathers of all infants had significantly less variance for several estimates of genetic similarity to the mothers compared with random males at both MHC loci. Additionally, the MHC diversity of these fathers was significantly higher than random males. We also found support for choice based on specific alleles; compared with random males, *Rhro-DQA1^∗^ 05* and *Rhro-DQB1^∗^ 08* were more common in both the OMU (one-male unit) males and the genetic fathers of offspring. This study provides new evidence for female mate choice for MHC-intermediate dissimilarity (rather than maximal MHC dissimilarity) and highlights the importance of incorporating multiple MHC loci and social structure into studies of MHC-based mate choice in non-human primates.

## Introduction

Females usually invest more in each individual offspring than males and thus tend to be the choosier sex when determining mating partners (Searcy, 1982; Andersson, 1994; Kokko et al., 2003). Female mate choice can occur before, during and after mating. Females may choose males that offer them “direct” material benefits such as food, high-quality territories or paternal care that can translate into increased reproductive success. Alternatively, females may choose males that offer “indirect” genetic benefits such as “disassortative mating” and “good-genes-as-heterozygosity” (Trivers, 1972; Andersson, 1994; Tregenza and Wedell, 2000; Moller and Jennions, 2001; Kokko et al., 2003; Neff and Pitcher, 2005; Kempenaers, 2007). “Disassortative mating” requires females to choose genetically dissimilar males by self-reference to their own genotypes in order to produce an excess of heterozygous offspring (Penn and Potts, 1999; Tregenza and Wedell, 2000; Mays and Hill, 2004). “Good-genes-as-heterozygosity” posits that females choose heterozygous males and can also result in the production of increased proportions of heterozygous offspring (Mays and Hill, 2004).

Several studies have examined specific functional loci effects and genome-wide effects of female mate choice. The genetic target(s) of female choice in most species thus generally remains unclear (Ferrandiz-Rovira et al., 2016). In most vertebrates, the highly polymorphic genes of the major histocompatibility complex (MHC) are key components of the immune system. These genes are involved in the recognition and presentation of intracellular (such as viruses) and extracellular (such as bacteria) antigens to T-cells, thereby contributing to a host immune response to pathogen attack (Doherty and Zinkernagel, 1975; Bernatchez and Landry, 2003). Several studies have shown that MHC genes are important for mate choice in several vertebrate species, with females not only benefiting directly by mating with “healthy” males (i.e., females may be less likely to be infected with pathogens from their mates) but also indirectly by producing offspring with enhanced capacity to fight pathogen infection (Landry et al., 2001; Richardson et al., 2005; Santos et al., 2017; Rekdal et al., 2019). MHC genes thus make an ideal study system to enable the identification of specific functional loci effects of female mate choice (Penn and Potts, 1999; Milinski, 2006; Schwensow et al., 2008a, b; Wedekind and Evanno, 2010).

A role for the MHC in mate choice has been found in a variety of vertebrate taxa (such as fish, reptiles, birds, rodents, and primates; Miller et al., 2009; Setchell et al., 2011; Evans et al., 2012; Paula Cutrera et al., 2012; Rekdal et al., 2019). There are three not necessarily mutually exclusive mechanisms by which MHC is relevant to mate choice. (1) Choice for dissimilarity by which one individual chooses a mating partner of a dissimilar MHC genotype. The benefits accrued are increased offspring heterozygosity with enhanced immunocompetence, and inbreeding avoidance (*Ailuropoda melanoleuca*, Zhu et al., 2019; *Microcebus murinus*, Huchard et al., 2013). Choice for intermediate dissimilarity is similar to choice for dissimilarity but is less pronounced. The chooser will benefit by producing offspring of intermediate rather than maximal MHC genetic diversity. This avoids any costs associated with excessive outbreeding depression, and/or locally adapted gene complexes affecting the immune systems of offspring being disrupted, and also avoids the consumption of mature T-cell repertoire in offspring with high MHC diversity (*Luscinia svecica*, Rekdal et al., 2019; *Mandrillus sphinx*, Setchell et al., 2016). (2) Choice for heterozygosity (diversity), in which heterozygous mating partners are preferred. Choosers benefit by associating with individuals with enhanced disease resistance and hence reduced infection, and by producing heterozygous offspring in variable environments (*Oceanodroma leucorhoa*, Hoover et al., 2018; *Microcebus murinus*, Schwensow et al., 2008a). (3) Choice for specific alleles in mating partners. Choosers can obtain benefits from associating with high-quality mates (such as reduced risk of contracting infection and higher intrasexual competitive ability) and produce more offspring with specific “good genes” (*Ctenomys talarum*, Paula Cutrera et al., 2012; *Cheirogaleus medius*, Schwensow et al., 2008b). Importantly, a lack of MHC-based mate choice in some species suggests that benefits associated with MHC-loci do not occur or may be context dependent (*Peromyscus californicus*, Melendez-Rosa et al., 2018; *Papio ursinus*, Huchard et al., 2010a).

The golden snub-nosed monkey (*Rhinopithecus roxellana*) is a good model system for investigating female mate choice, because this endangered primate species lives in a multi-level society (MLS). Each breeding band of up to 100 individuals is comprised of several one-male units (OMUs), each made up of a single adult male, and several adult females and their juvenile and infant offspring. The breeding band is followed by an all-male band (AMB), a group of former resident males, young males who have reached sexual maturity, and sub-adult males who have been ejected from the breeding band (Qi et al., 2014). These AMB males often challenge and attempt to usurp the OMU males and/or opportunistically engage in extra-pair copulations with adult females (Grueter et al., 2017; Qi et al., 2017). The *R. roxellana* social structure thus provides adult females with opportunities to exhibit mate choice between their own OMU male and the other adult males within the breeding band, and between their own OMU male and AMB males. In addition to the social status and competitiveness of adult males, female mate choice may play a major role in the usurpation of OMU males by AMB males (Qi et al., 2009; Fang et al., 2018). Moreover, previous field observations have found that females usually initiate extra-pair copulations when their OMU males are not present. Indeed, approximately 50% of offspring are fathered by a male other than the adult male of the same OMU as the mother (Guo et al., 2010; Qi et al., 2020). Female *R. roxellana* may also preferentially engage in extra-pair copulations with those males who have recently achieved OMU “leader” male status (Zhao et al., 2005; Qi et al., 2020).

Because female *R. roxellana* have ample opportunities to express their mating preferences in different ways, the high rates of extra-pair paternity in populations may not be the result of a single mechanism. We therefore tested alternative genetic mechanisms underlying female mate choice in *R. roxellana* using 3 years of social organization and genetic (20 microsatellites and two MHC loci: *DQA1* and *DQB1*) data. We made four *priori* predictions. (1) Females are likely to be members of OMUs headed by males based on the genetic traits of the OMU leader male, consistent with “choice for specific alleles” and “choice for heterozygosity.” (2) Females use their own MHC genetic characteristics as a reference on which to choose the fathers of their offspring, and they will choose males who are most genetically different to themselves (“choice for dissimilarity”). (3) The MHC and/or microsatellite genetic diversity of males will affect the probability of a female producing offspring with her own OMU leader male or via extra-pair copulations. (4) MHC and/or microsatellite genetic diversity and compatibility will affect the likelihood of OMU males having infants present within their OMU that they have not fathered [extra-pair offspring (EPO)].

## Materials and Methods

### Study Site, Reproductive Data and Sample Collection

Field work was undertaken in the Zhouzhi National Nature Reserve (ZNNR), which is located on the northern edge of the Qinling Mountains (33°48′68″N, 108°16′18″E), Shaanxi Province, central China [see Li et al. (2000) and Li and Zhao (2007) for details]. Two *R. roxellana* troops are present in this area (East Ridge Troop: ERT and West Ridge Troop: WRT) (Li et al., 2000). We used the WRT for this study, which during the 3 years study period consisted of a breeding band of 10–13 one-male units (∼130–150 individuals) and one all-male band (∼ 40 individuals). These monkeys have been provisioned food every winter since October 2001 to enable close observation and recognition of individuals. Individual identification was made according to physical characteristics described previously (Qi et al., 2014).

Data were collected from September 2015 until May 2017. Data on the composition of the study troop OMUs were collected by focal animal sampling (Altmann, 1974) during the annual peak mating period (from September to December). The current fertility status of each female was determined from the subsequent breeding period (from March to May). Fecal and hair samples were collected non-invasively from 145 individuals. We sampled 80–90% of all individuals from the breeding band, samples that consisted of all adult OMU males (*N* = 16), all females that produced infants during the study period (*N* = 48), and all infants produced during the study period (*N* = 54). We also sampled approximately 70% of all AMB individuals, sampling that consisted of all adult (*N* = 21) and sub-adult males (*N* = 6). Juveniles were excluded because they cannot reproduce. In 2015, there were in total 20 mother-infant pairs. However, insufficient sampling for four infants prevented adequate PCR amplification for inclusion in the dataset. Therefore, only 16 mother-infant pairs were used for the subsequent data analysis. Samples from the infants, which were all born from 2015 to 2017, were collected by maternal-infant pairing (the mother-infant relationship is initially determined by the fact that infants are carried and fed by their mothers). All individuals were sampled repeatedly at least twice to ensure the accuracy of the genetic data.

### DNA Extraction and Genotyping

DNA from hair follicles and fecal samples were extracted following methods described by Allen et al. (1998), and using QIAamp PowerFecal DNA Kits (QIAGEN, Germany), respectively.

We used 20 highly polymorphic microsatellite loci (D10s1432; D10s2483; D10s676; D12s375; D14s306; D18s1371; D19s1034; D19s582; D20s206; D21s2054; D3s1766; D6s1036; D6s501; D7s1804; D7s2204; D7s820; D8s1049; D9s252; D9s905; D6s49) to conduct paternity analysis for all 54 infants born from 2015 to 2017. The polymerase chain reaction (PCR) and the analysis of the microsatellite loci were conducted using an extended dataset, with the primers and conditions as described by Huang et al. (2016).

We genotyped 54 parent-offspring triads and adult and sub-adult males from the AMB (*N* = 27) at the highly polymorphic *Rhro-DQA1* and *Rhro-DQB1* exon 2 sequences. The primers, PCR amplifications, and genotyping were conducted using methods as previously described (Zhang et al., 2016).

### Data Analysis

#### Parentage Analysis

For all infants, from whom information about their mothers and OMU males was available from field work, paternity analysis was performed using the software CERVUS 3.0.6 (Kalinowski et al., 2007), based on the 20 microsatellites. MHC genotype data concurred with the parentage results according to Mendelian inheritance.

Using parentage analysis, we categorized each adult male to one of the following two groups:

(i) OMU male: the resident male of the same OMU as the mother of each infant during the previous mating season.
(ii) Genetic father: the real father of each infant based on the result of parentage analysis. This could have been the resident OMU male of the same OMU as the mother, the resident male of a neighboring OMU to that of the mother, or an AMB male.

Using parentage analysis, we categorized each infant to one of the following two groups:

(i) Extra-pair offspring (EPO): an infant fathered by a male who was not the infant’s OMU male.
(ii) Within-pair offspring (WPO): an infant fathered by its OMU male.

#### Test Parameters for Hypotheses

To test for mate choice based on MHC-dissimilarity, we estimated the following parameters: (1) the genetic similarity of MHC alleles between the individuals of adult male-female pairs (*D*_*FM*_), calculated as *D*_*FM*_ = 2*N*_*FM*_/(*N*_*F*_ + *N*_*M*_), where *N*_*F*_ and *N*_*M*_ are the number of MHC alleles in female and male, and *N*_*FM*_ is the number of these alleles shared by both individuals (Wetton et al., 1987); (2) the pairwise evolutionary amino acid distance (*E*_*aadis*_), which was calculated as *E*_*aadis*_ = *E*_*AB*_ + *E*_*Ab*_ + *E*_*aB*_ + *E*_*ab*_, where A, a, B, b represent the four alleles carried by the female and the male (Landry et al., 2001). The evolutionary distance between two alleles is calculated by MEGA 7.0 (Kumar et al., 2016); and (3) the pairwise functional amino acid distance (*F*_*aadis*_). The physiochemical properties of each amino acid were represented by five z-descriptors: z1 (hydrophobicity), z2 (steric bulk), z3 (polarity), and z4 and z5 (electronic effects) (Sandberg et al., 1998). A matrix between alleles was constructed to enable the Euclidian distance of all amino acids to be calculated (Huchard et al., 2013). The formula used to calculate *F*_*aadis*_ is similar to that used for *E*_*aadis*_, as*F*_*aadis*_ = *F*_*AB*_ + *F*_*Ab*_[*cpsbreak*] + *F*_*aB*_ + *F*_*ab*_.

To test for mate choice based on MHC-heterozygosity, we calculated the MHC heterozygosity (*H*_*MHC*_) of all adult OMU and AMB males (*DQA1* and *DQB1*). We used 0 to represent homozygotes and 1 to represent heterozygotes for each MHC locus. To test for mate choice based on MHC-diversity, two parameters were used: (1) the evolutionary amino acid distance (intra-*E*_*aadis*_) and (2) the functional amino acid distance (intra-*F*_*aadis*_) between two alleles within a locus for each adult OMU and AMB male.

To test for mate choice based on specific MHC genes, the MHC allele frequency (*AF*) of each locus in adult OMU and AMB males was used.

To test for mate choice based on overall heterozygosity, we calculated the microsatellite multi-locus heterozygosity in adult OMU and AMB males (*MLH*, the number of heterozygous microsatellite loci as a proportion of the total number of typed loci).

To test for inbreeding avoidance, we calculated Queller and Goodnight’s (1989) relatedness *r* using the program SPAGeDi v1.5a (Hardy and Vekemans, 2002) to quantify the genetic relatedness of each actual mother of each infant to each OMU and AMB male combination (Table 1).

**TABLE 1:** Summary table of the test parameters for alternative hypotheses.

Mate choice based on	Parameter	
MHC-dissimilarity	*D*_*FM*_	the genetic similarity of MHC alleles between females and males
	*E*_*aadis*_	the pairwise evolutionary amino acid distance between the MHC alleles of mates
	*F*_*aadis*_	the pairwise functional amino acid distance between the MHC alleles of mates
MHC-heterozygosity (diversity)	*H*_*MHC*_	MHC heterozygosity of individual male
	intra-*E*_*aadis*_	the evolutionary amino acid distance between two alleles in males
	intra-*F*_*aadis*_	the functional amino acid distance between two alleles in males
specific MHC genes	*AF*	the MHC allele frequency of each locus in males
overall heterozygosity	*MLH*	the microsatellite multi-locus heterozygosity in males
inbreeding avoidance	*r*	the genetic relatedness between females and males

#### Randomization Tests

We conducted a randomization test based on Monte Carlo sampling to investigate three non-exclusive hypotheses on MHC-based mate choice. We simulated a random model of female choice by letting each female randomly select 10 000 times between all accessible adult males of the corresponding year. We then compared the observed mean value of OMU males or genetic fathers, to the simulated range of randomly “selected” males. We calculated *P*-values as the proportion of the total number of iterations greater or smaller than the observed mean. Observed values were significant if they fell outside of the 97.5–2.5% confidence interval (CI). If females prefer males with heterozygous or specific alleles as OMU males, we expected the mean of *H*_*MHC*_, intra-*E*_*aadis*_, intra-*F*_*aadis*_, *MLH*, and *AF* for the OMU males would to be significantly higher than that of randomly assigned males. If females choose males based on their own MHC genotype as the genetic fathers of their offspring, A lower mean of *D*_*FM*_ and a higher mean of *E*_*aadis*_, *F*_*aadis*_ for the genetic fathers to mothers than random suggests mate choice for maximum MHC-dissimilarity. However, the mean value for mate choice for intermediate MHC-dissimilarity cannot be distinguished from that for random mating. Therefore, in order to compare the variance of 54 observed mates with random mates, we produced 54 randomly selected “mates” and repeated the process 10,000 times to calculate the distribution of 10,000 variances. If the observed variance of pairwise MHC-dissimilarity is significantly lower than the random variance distribution, then this is consistent with mate choice for intermediate MHC-dissimilarity (Forsberg et al., 2007). If females are attempting to avoid the costs of inbreeding, we expected the mean of *r* for the genetic fathers and mothers to be significantly higher than that for random mating.

#### Extra-Pair Copulation

To test if patterns of extra-pair copulation were consistent with female choice for MHC-based and/or genome-wide effects, we used a series of generalized linear mixed models (GLMM) to measure the probability of a male being an OMU male of an EPO or the genetic father of an EPO. Each model used a single explanatory measure. This was one of three female–male MHC-dissimilarity measures (*D*_*FM*_, pairwise *E*_*aadis*_, pairwise *F*_*aadis*_), one of three MHC-heterozygosity (diversity) measures for males (*H*_*MHC*_, intra-*E*_*aadis*_, intra-*F*_*aadis*_), the overall heterozygosity parameter of males (*MLH*), or the genetic relatedness between the female (mother) and either the OMU male of an EPO or the genetic father of an EPO (*r*). Each model used a binary response variable defined as 1 for an OMU male with an extra-pair offspring (EPO) or 0 for a genetic father of an EPO. To remove the effects of data pseudo-replication, female ID, male ID, and sampling year were all included as random factors.

We also used generalized linear mixed models (GLMM) to test for the effects of variation in genetic measurements (*H*_*MHC*_, intra-*E*_*aadis*_, intra-*F*_*aadis*_, *D*_*FM*_, pairwise *E*_*aadis*_, pairwise *F*_*aadis*_, *MLH*, and *r*) on the probability of individual offspring being the product of an extra-pair copulation. We used a binary response variable defined as 1 for an OMU male with an extra-pair offspring (EPO) or 0 for an OMU male with a within-pair offspring (WPO). To remove the effects of pseudo-replication, female ID, male ID, and sampling year were all included as random factors.

To account for any possible false discovery regarding multiple tests, we applied Bonferroni corrections (*n* = 8; *p*-threshold = 0.006). All GLMM analyses were conducted with R 3.6.1. (R-Core Team, 2019), either using the base package or with the additional package “lmerTest” (Kuznetsova et al., 2017).

## Results

### Microsatellite Typing and Paternity Analysis

A total of 145 individuals were typed using 20 microsatellite loci and 54 parent-offspring triads were identified. For all infants, information about their mothers and OMU males was determined from behavioral observations. Thirty-three infants (61%) were fathered by their OMU male, the same male that headed their mother’s OMU. The remaining 21 infants (39%) were the result of extra-pair copulations. Of these 21 infants, 14 (26% of all infants) were fathered by an OMU adult male from a different OMU to that of their mother, with 7 (13% of all infants) being fathered by an AMB male (Supplementary Table 1).

### MHC Typing

The *DQA1* locus genotypes were determined for 144 individuals (16 OMU males, 27 AMB males, 48 adult females, and 53 infants) and the *DQB1* locus genotypes for 145 individuals (16 OMU males, 27 AMB males, 48 adult females, and 54 infants). For both *DQA1* and *DQB1* loci, we identified five previously reported alleles: *Rhro-DQA1^∗^01*, *02*, *03*, *05*, and *08*, GenBank: JQ217107-JQ217115; *Rhro-DQB1^∗^01*, *04*, *08*, *09*, and *17*, GenBank: JQ217116-JQ217131, KU184585 (Luo et al., 2012; Zhang et al., 2016).

There was no significant correlation between MHC heterozygosity and multi-locus heterozygosity. [*H*_*MHC*_-*DQA1* vs. *MLH* (*N* = 43): Spearman’s rho = −0.061, *p* = 0.697; *H*_*MHC*_-*DQB1* vs. *MLH* (*N* = 43): Spearman’s rho = −0.065, *p* = 0.680]. Our estimates of pairwise MHC genetic similarity did not correlate with relatedness as estimated from microsatellites [*D*_*FM*_-*DQA1* vs. *r* (*N* = 52): Spearman’s rho = −0.045, *p* = 0.753; *D*_*FM*_-*DQB1* vs. *r* (*N* = 52): Spearman’s rho = 0.017, *p* = 0.906].

### Female Choice for OMU Males

**FIGURE 1:**
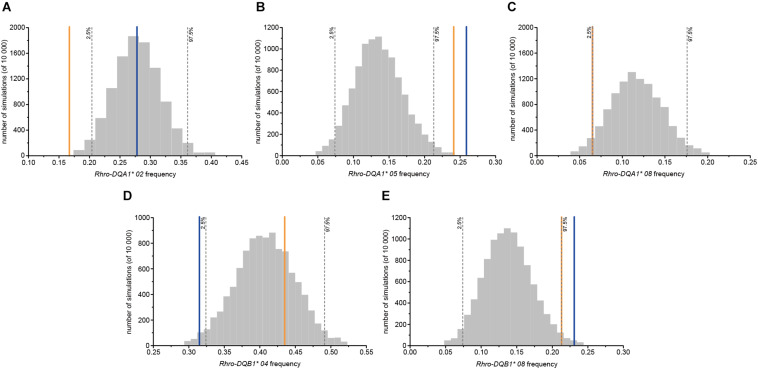
Frequencies of MHC alleles for OMU males, genetic fathers, and randomly assigned males. Blue solid line: OMU males; orange solid line: genetic fathers; gray bars: frequency distributions of mean values generated from 10,000 simulations of random males. Two-tailed 95% confidence intervals are indicated by dashed lines which displayed cut-offs for significant departures from random mating. **(A)** Rhro-DQA1* 02, **(B)** Rhro-DQA1* 05, **(C)** Rhro-DQA1* 08, **(D)** Rhro-DQB1* 04, and **(E)** Rhro-DQB1* 08.

No evidence of female choice for OMU males based on MHC dissimilarity, intermediate dissimilarity, heterozygosity, and diversity was detected at both loci (Table 2). However, the frequency of *Rhro-DQA1^∗^ 05* and *Rhro-DQB1^∗^ 08* for OMU males was significantly higher than for randomly assigned males, and significantly lower frequencies of *Rhro-DQA1^∗^ 08* and *Rhro-DQB1^∗^ 04* (Table 2 and Figure 1).

**TABLE 2:** Results of randomization tests for differences in genetic parameters (MHC genes and microsatellites).

Hypotheses	Parameter	OMU males vs. randomly assigned males		Genetic fathers vs. randomly assigned males	
		*DQA1*	*DQB1*	*DQA1*	*DQB1*
MHC dissimilarity	*D*_*MF*_	obs = 0.407	obs = 0.426	obs = 0.420	obs = 0.497
		sim = 0.478[0.395, 0.568]	sim = 0.463[0.370, 0.559]	sim = 0.478[0.395, 0.568]	sim = 0.463[0.370, 0.559]
	*E*_*aadis*_	obs = 0.497	obs = 0.466	obs = 0.517	obs = 0.463
		sim = 0.506[0.463, 0.549]	sim = 0.491[0.447, 0.534]	sim = 0.506[0.463, 0.549]	sim = 0.491[0.447, 0.534]
	*F*_*aadis*_	obs = 174.8	obs = 183.8	obs = 182.6	obs = 182.6
		sim = 180.0[164.2, 195.4]	sim = 193.7[176.4, 210.4]	sim = 180.0[164.2, 195.4]	sim = 193.7[176.4, 210.4]
MHC-heterozygosity	*H*_*MHC*_	obs = 0.796	obs = 0.704	obs = 0.852	obs = 0.778
		sim = 0.833[0.741, 0.926]	sim = 0.741[0.630, 0.852]	sim = 0.833[0.741, 0.926]	sim = 0.741[0.630, 0.852]
(diversity)	intra-*E*_*aadis*_	obs = 0.162	obs = 0.133	obs = 0.181	obs = 0.155*
		sim = 0.155[0.127, 0.182]	sim = 0.129[0.102, 0.155]	sim = 0.155[0.127, 0.182]	**sim = 0.129[0.102, 0.155]**
	intra-*E*_*aadis*_	obs = 57.45	obs = 52.32	obs = 64.56	obs = 61.21*
		sim = 55.60[45.20, 65.34]	sim = 51.06[40.43, 61.17]	sim = 55.60[45.20, 65.34]	**sim = 51.06[40.43, 61.17]**
specific MHC genes	*AF*	*01*, obs = 0.315	*01*, obs = 0.102	*01*, obs = 0.426	*01*, obs = 0.102
		sim = 0.380[0.306, 0.463]	sim = 0.102[0.046, 0.167]	sim = 0.380[0.306, 0.463]	sim = 0.102[0.046, 0.167]
		*02*, obs = 0.278	*04*, obs = 0.315*	*02*, obs = 0.167**	*04*, obs = 0.435
		sim = 0.278[0.204, 0.361]	**sim = 0.407[0.324, 0.491]**	**sim = 0.278[0.204, 0.361]**	sim = 0.407[0.324, 0.491]
		*03*, obs = 0.083	*08*, obs = 0.231**	*03*, obs = 0.102	*08*, obs = 0.213*
		sim = 0.083[0.037, 0.130]	**sim = 0.139[0.074, 0.213]**	sim = 0.083[0.037, 0.130]	**sim = 0.139[0.074, 0.213]**
		*05*, obs = 0.259**	*09*, obs = 0.093	*05*, obs = 0.241**	*09*, obs = 0.093
		**sim = 0.139[0.074, 0.213]**	sim = 0.139[0.074, 0.213]	**sim = 0.139[0.074, 0.213]**	sim = 0.139[0.074, 0.213]
		*08*, obs = 0.065*	*17*, obs = 0.259	*08*, obs = 0.065*	*17*, obs = 0.157
		**sim = 0.111[0.065, 0.176]**	sim = 0.204[0.139, 0.287]	**sim = 0.111[0.065, 0.176]**	sim = 0.204[0.139, 0.287]
		**SSR**			
overall heterozygosity	*MLH*	obs = 0.589		obs = 0.578	
		sim = 0.582[0.551, 0.612]		sim = 0.582[0.551, 0.612]	
inbreeding avoidance	*r*	obs = −0.000		obs = −0.010	
		sim = 0.002[−0.052, 0.059]		sim = 0.002[−0.052, 0.059]	

Statistical significance is indicated in bold. obs, observed mean; sim, simulated mean [95% CI]. *P* ≤ 0.05 in the statistical tests is indicated with *; *P* ≤ 0.01 in the statistical tests is indicated with **.

For female mate choice based on genome-wide effects, we found no difference between OMU males and randomly assigned males for microsatellite multi-locus heterozygosity (*MLH*), or relatedness (Table 2).

### Female Choice for Genetic Fathers

**FIGURE 2:**
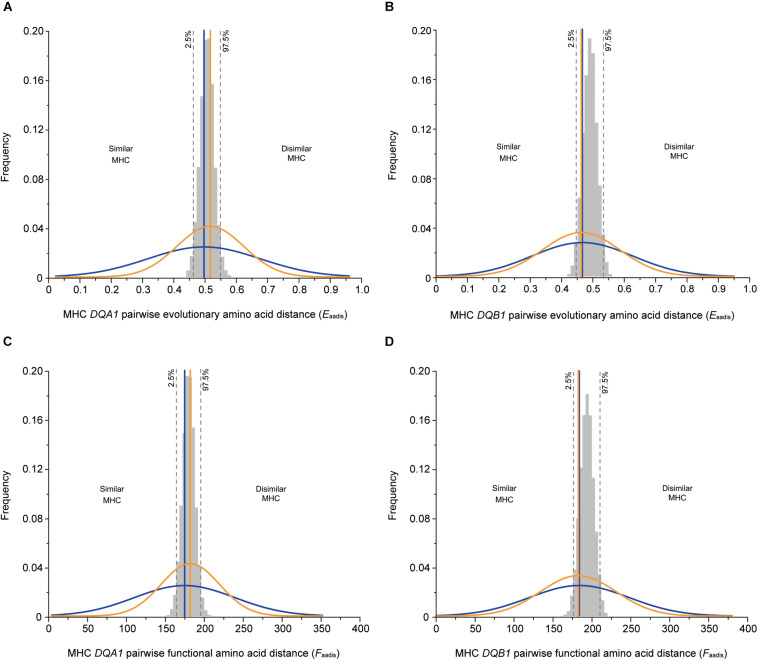
MHC genetic similarity parameters between male and female for OMU males, genetic fathers and randomly assigned males. The observed values for the OMU males/genetic fathers and female are visualized as normalized curves in blue and orange. Blue solid line: mean of OMU males; orange solid line: mean of genetic fathers; gray bars: frequency distributions of mean values generated from 10,000 simulations of random mates. Two-tailed 95% confidence intervals are indicated by dashed lines which displayed cut-offs for significant departures from random mating. **(A,B)** pairwise evolutionary amino acid distance, **(C,D)** pairwise functional amino acid distance. **(A,C)** at *DQA1* locus, **(B,D)** at *DQB1* locus.

The mean values of the genetic fathers-females did not significantly differ to the mean values for randomly assigned males-females in terms of *D*_*FM*_, *E*_*aadis*_, and *F*_*aadis*_ (Table 2 and Figure 2). However, consistent with the intermediate dissimilarity hypothesis, variance for *E*_*aadis*_, and *F*_*aadis*_ of the genetic fathers-females were significantly lower than the corresponding values for randomly selected mates at both loci (Table 3).

**TABLE 3:** Results of randomization tests between OMU males/genetic fathers and randomly assigned males in measures of variance in MHC dissimilarity parameter.

Parameter	OMU males vs. randomly assigned males		Genetic fathers vs. randomly assigned males	
	*DQA1*	*DQB1*	*DQA1*	*DQB1*
variance in *D*_*MF*_	obs = 0.095 sim = 0.111[0.073, 0.188]	obs = 0.095 sim = 0.132[0.081, 0.224]	obs = 0.087 sim = 0.111[0.073, 0.188]	obs = 0.128 sim = 0.132[0.081, 0.224]
variance in *E*_*aadis*_	obs = 0.032 sim = 0.028[0.015, 0.043]	obs = 0.025 sim = 0.029[0.017, 0.042]	obs = 0.011** sim = 0.028[0.015, 0.043]	obs = 0.015* sim = 0.029[0.017, 0.042]
variance in *F*_*aadis*_	obs = 4137.9 sim = 3626.2[1936.0, 5561.6]	obs = 4111.1 sim = 4421.0[2591.5, 6492.8]	obs = 1394.9** sim = 3626.2[1936.0, 5561.6]	obs = 2345.8* sim = 4421.0[2591.5, 6492.8]

Statistical significance is indicated in bold. obs, observed mean; sim, simulated mean [95% CI]. *P* ≤ 0.05 in the statistical tests is indicated with *; *P* ≤ 0.01 in the statistical tests is indicated with **.

**FIGURE 3:**
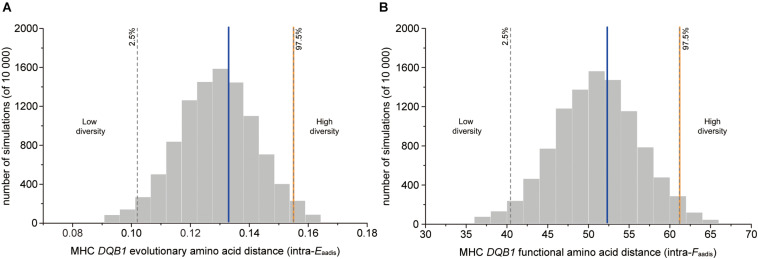
MHC genetic diversity parameters for OMU males, genetic fathers and randomly assigned males. Blue solid line: OMU males; orange solid line: genetic fathers; gray bars: frequency distributions of mean values generated from 10,000 simulations of random males. Two-tailed 95% confidence intervals are indicated by dashed lines which displayed cut-offs for significant departures from random mating. **(A)** evolutionary amino acid distance of individual MHC alleles (intra-*E*_*aadis*_), **(B)** functional amino acid distance of individual MHC alleles (intra-*F*_*aadis*_).

The genetic fathers had significantly higher value of evolutionary amino acid distances (intra-*E*_*aadis*_) and functional amino acid distances (intra-*F*_*aadis*_) than randomly assigned males at *DQB1* (Table 2 and Figure 3).

The choice of specific MHC alleles for the genetic fathers was largely consistent with that for the OMU males. Both had significantly higher frequencies of *Rhro-DQA1^∗^ 05* and *Rhro-DQB1^∗^ 08* than randomly assigned males, but significantly lower frequencies of *Rhro-DQA1^∗^ 02* and *Rhro-DQA1^∗^ 08* (Table 2 and Figure 1).

For choice based on genome-wide effects, we found no difference between genetic fathers and randomly assigned males for microsatellite multi-locus heterozygosities (*MLH*). The relatedness values for the genetic fathers and females did not differ to those of randomly assigned mates (Table 2).

### Extra-Pair Paternity

We found no significant effect of each genetic variable on the probability of one male being an OMU male of an EPO or a genetic father of an EPO (GLMM; Supplementary Tables 2–9). Furthermore, we found no significant effect of each genetic variable on the probability of individual offspring being the product of an extra-pair copulation (GLMM; Supplementary Tables 10–17).

## Discussion

Although *R. roxellana* is generally regarded as being polygynous, its mating system is dynamic and is likely to change in response to various factors such as OMU take-overs and female transfers between different OMUs (Zhang et al., 2006; Qi et al., 2009). Moreover, our new parentage analysis showed a 39% rate of extra-pair paternity, which broadly concurs with the high rates in the same *R. roxellana* population in different years (57% in both 2005 and 2014–2015; Guo et al., 2010; Qi et al., 2020). Our results suggest that female *R. roxellana* choose the genetic fathers of their offspring non-randomly. Our data are consistent with female preference for mates of intermediate MHC dissimilarity and high MHC diversity, and showed evidence of choice for specific MHC genes. The measured values of MHC dissimilarity between females and the genetic fathers of their offspring was consistently close to the mean value of the random model, with a significantly reduced variance. The difference between individual MHC alleles of the genetic fathers of offspring was also significantly higher than for the randomly chosen males. In both the mothers’ OMU males and the genetic fathers of offspring, the frequency of *Rhro-DQA1^∗^ 05* and *Rhro-DQB1^∗^ 08* was significantly higher than in randomly assigned males, and the frequency of *Rhro-DQB1^∗^ 08* was significantly lower than in randomly assigned males. Finally, our microsatellite data showed no evidence of choice for genome-wide heterozygosity advantage or inbreeding avoidance. Genes of the MHC or microsatellites convey different information, suggesting that the trends we observed regarding MHC class II genes were not due to genome-wide effects. The functional loci of the MHC are thus more likely to be the targets of female mate choice in *R. roxellana*.

Several studies have also reported evidence for intermediate dissimilarity choice at MHC loci (Bonneaud et al., 2006; Forsberg et al., 2007; Eizaguirre et al., 2009; Rekdal et al., 2019). This concurs with mate choice for MHC dissimilarity to produce offspring that can cope with a wide-range of pathogens. However, it is noteworthy that at some point the costs of choice for MHC genes will outweigh the benefits due to: (1) self-reactive autoimmune responses, (2) the cost of coadapted gene complex disruption, and (3) a consumption of mature T-cell repertoire in individual offspring with high MHC diversity (Nowak et al., 1992; Hendry et al., 2000; Woelfing et al., 2009). This concurs with the hypothesis that the optimal MHC diversity for offspring could be intermediate rather than maximal due to a trade-off between individual MHC alleles (Kalbe et al., 2009; Woelfing et al., 2009). Some experimental studies on infection by multiple parasites and pathogens have shown that high rates of MHC polymorphism may reduce the immune ability of individual hosts (Wegner et al., 2003; Ilmonen et al., 2007). Females may thus choose males with intermediate differences in genotypes as fathers of their offspring to avoid producing offspring with high MHC polymorphism. However, we found no evidence that any of the genetic variables in this study affected the probability of extra-pair copulation, and no evidence that any of the genetic variables affected the likelihood of OMU males fathering EPOs. This suggests that within the scope of our research, any benefits to females via extra-pair copulations may not be entirely genetic. In *R. roxellana*, extra-pair copulations are mostly initiated by females and are not confined to the mating season (Zhao et al., 2005). Extra-pair copulations may thus not always serve a direct reproductive function and may benefit females in other ways. For example, extra-pair copulations may help females establish a beneficial social relationship with a male (Lemasson et al., 2008). In *R. roxellana* multiple females from one OMU have been recorded engaging in extra-pair copulations with the same AMB male, which has successfully usurped the OMU male during the course of their pregnancies (Qi et al., 2020). Female *R. roxellana* may also produce extra-pair offspring as an infanticide avoidance strategy via paternity confusion (Qi et al., 2020). Female initiated extra-pair copulations are thus unlikely to be solely driven by genetic benefits to females.

Both human and non-human primate studies have shown evidence of mate choice for MHC-dissimilarity and diversity, with fewer reports of mate choice for intermediate MHC dissimilarity, optimal diversity, or specific MHC genes (Winternitz and Abbate, 2015). To date, there are relatively few reports of mate choice for specific MHC genes in non-human primates (Sauermann et al., 2001; Schwensow et al., 2008a, b; Setchell et al., 2009a, 2013, 2016; Huchard et al., 2010a, 2013; Yang et al., 2014; Sterck et al., 2017). In fat-tailed dwarf lemurs (*Cheirogaleus medius*), male and female formed life-long breeding pairs but with high rate of extra-pair paternity (44%), social males have higher frequency of *MHC-DRB* supertype S1 (20 MHC supertypes defined by hierarchical clustering based on amino acid sequence in the 50 different MHC-DRB alleles) than random males (Schwensow et al., 2008b). In mandrills (*Mandrillus sphinx*), a species that exhibits male mate-guarding of females, males prefer to guard females who do not possess *MHC-DRB* supertype S1, an MHC supertype that is associated with decreased immune function in the study population (Setchell et al., 2016).

In another *R. roxellana* population, Yang et al. (2014) found no evidence for female mate choice based on MHC genotypes. Our study used similar statistical methods in order to quantify the effects of MHC variation on mate choice, as well as comparisons between real and random males. However, there are several differences between this present study and that of Yang et al. (2014). We found that both *DQA1* and *DQB1* loci of the MHC II gene significantly affected mate choice, whereas Yang et al. (2014) used the *DRB* locus of the MHC II gene and found no significant effects. Although any functional differences among the *DQA1*, *DQB1*, and *DRB* loci remain unknown for *R. roxellana*, there may be different mate choice selection pressures on different loci. For instance, in gray mouse lemurs (*Microcebus murinus*), disassortative mate choice was detected at *DRB* but not at *DQB* (Huchard et al., 2013).

An alternative explanation for the discrepancy between our new results and those of Yang et al. (2014) may be due to insufficient sample size, resulting in type II errors in the interpretation of highly polymorphic MHC genes (Hoover and Nevitt, 2016). The breeding band studied by Yang et al. (2014) was smaller than our study breeding band and contained only five adult males (four OMU leader males and a single AMU male) and 15 adult females. Moreover, Yang et al. (2014) also found extra-pair paternity, but only less than 10% of all infants were the result of an extra-pair copulation. Due to the larger number of adult males per female in the breeding band of our study, females thus had more opportunities to exhibit mate-choice and/or extra-pair copulation. In order to minimize conflict between adult males, interactions between the AMB and OMUs of the population studied by Yang et al. (2014) had been experimentally restricted so females would have had reduced opportunities for expressing their mate preferences (Qi et al., 2020). Studies of a wide variety of different taxa, including non-human primates, have shown that female mate choice is usually dependent on ecological conditions that alter the costs and benefits of being choosy (Andersson, 1994). For example, when dominant males are able to prevent other males from obtaining mating opportunities, such as in small groups, female mating preferences are constrained (Setchell and Huchard, 2010).

Major histocompatibility complex-based mate choice in *R. roxellana* posits the question: which phenotypic signals do females use to evaluate the genetic quality of potential mates? In some non-human primates, MHC profiles have been shown to correlate with variation in secondary sexual traits in both sexes. In Chacma baboons (*Papio ursinus*), the specific MHC supertype (S1) is associated with female physical condition and the size of sexual swellings, even there is no evidence of male mate choice for MHC dissimilarity, diversity or rare MHC genotypes (Huchard et al., 2010a, b). Female sexual swellings are a reliable indicator of female reproductive condition to males, and males preferring females with larger swellings (Huchard et al., 2009). In Mandrills (*Mandrillus sphinx*), male facial redness which were preferred by females, were positively correlated with MHC supertypes S4 and S11 (Setchell, 2005; Setchell et al., 2009b). *R. roxellana* exhibits several sexually dimorphic traits such as body size, facial color, and pelage color (Zhang et al., 2006). We speculate that females may assess male MHC quality using one or more of these traits. Additionally, olfactory cues to assess MHC dissimilarity may be involved, as have been found in diverse taxa such as sand lizards (*Lacerta agilis*), blue petrels (*Halobaena caerulea*), bank voles (*Myodes glareolus*), and humans (Jacob et al., 2002; Olsson et al., 2003; Thornhill et al., 2003; Radwan et al., 2008; Leclaire et al., 2017). In mandrills (*Mandrillus sphinx*), although male odors are not related to any specific MHC supertype, odor similarity is related to MHC similarity (Setchell et al., 2011).

Females may choose to mate with those extra-pair males that have a high likelihood of usurping an established OMU leader male, and may thus obtain future direct benefits from being a member of that male’s OMU (Qi et al., 2020). Female mate choice may thus play an important role in the social organization of *R. roxellana*. The *R. roxellana* multilevel society provides opportunities for females to choose mates at different levels. The potential for female mate choice to contribute to the maintenance of this multilevel society may thus vary accordingly. The lack of cultural, socioeconomic, and technological factors, along with superficial similarities in social structure, makes the study of such non-human primates a more productive alternative to current human populations (Winternitz and Abbate, 2015).

## Conclusion

To conclude, we investigated the effects of adaptive MHC genes and neutral microsatellites on female mate choice in *R. roxellana*. Our results are consistent with females basing their mate choice, at least in part, on benefits associated with intermediate MHC-dissimilarity, MHC-diversity, and specific MHC alleles but not on benefits associated with microsatellites. We emphasize that because we did not measure actual female mate choice in a behavioral context, our methods only enabled the evaluation of the effects of mate choice after choice had occurred (Andersson, 1994; Wagner, 1998; Qi et al., 2014). Finally, the differences in our results to those of another population of the same species emphasizes the importance of incorporating multiple MHC loci, large sample sizes, and variable social structures into studies of MHC-based mate choice in non-human primates.

## Data Availability Statement

The datasets presented in this study can be found in online repositories. The names of the repository/repositories and accession number(s) can be found below: https://www.ncbi.nlm.nih.gov/genbank/, JQ217107–JQ217115 and https://www.ncbi.nlm.nih.gov/genbank/, JQ217116–JQ217131.

## Ethics Statement

The animal study was reviewed and approved by the Ethics Committee of the College of Life Sciences, Northwest University.

## Author Contributions

PZ and B-GL designed the study and helped to draft the manuscript. B-YZ, H-YH, and C-MS carried out the molecular genetic studies. B-YZ performed the statistical analysis and drafted the manuscript. KH andDWDedited the manuscript. XY provided data support in response to comments. X-WW, H-TZ, and C-LW participated in the sampling. All authors read and approved the final manuscript.

## Conflict of Interest

The authors declare that the research was conducted in the absence of any commercial or financial relationships that could be construed as a potential conflict of interest.
