# Supplementary material for: MHC-Based Mate Choice in Wild Golden Snub-Nosed Monkeys
Source: Front Genet. 2020 Dec 21;11:609414. doi: 10.3389/fgene.2020.609414 (PMC7779673; doi:10.3389/fgene.2020.609414)
Supplement: Supplementary file 1 [file Data_Sheet_1.docx]

Supplementary Material

# Supplementary Tables

**Supplementary Table 1.** The parentage results for 54 infants born from 2015 to 2017.

| OMU male | Mother ID | Offspring ID | Genetic father | Comments |
| --- | --- | --- | --- | --- |
| BX | AF1 | BX-AF1-I15 | SQ | Neighbouring OMU male |
| BX | AF2 | BX-AF2-I15 | SQ | Neighbouring OMU male |
| G3 | AF1 | G3-AF1-I15 | G3 | OMU male |
| HB | AF1 | HB-AF1-I15 | HB | OMU male |
| HB | AF2 | HB-AF2-I15 | WF | AMU male |
| HB | AF3 | HB-AF3-I15 | WF | AMU male |
| KO | AF1 | KO-AF1-I15 | LD | Neighbouring OMU male |
| KO | AF2 | KO-AF2-I15 | SQ | Neighbouring OMU male |
| LD | AF1 | LD-AF1-I15 | TB | Neighbouring OMU male |
| SQ | AF1 | SQ-AF1-I15 | SQ | OMU male |
| SQ | AF2 | SQ-AF2-I15 | SQ | OMU male |
| SQ | AF3 | SQ-AF3-I15 | SQ | OMU male |
| ST | AF1 | ST-AF1-I15 | ST | OMU male |
| SX | AF1 | SX-AF1-I15 | SX | OMU male |
| WX | AF1 | WX-AF1-I15 | WX | OMU male |
| WX | AF2 | WX-AF2-I15 | WX | OMU male |
| BX | AF3 | BX-AF3-I16 | SQ | Neighbouring OMU male |
| G3 | AF1 | G3-AF1-I16 | G3 | OMU male |
| G3 | AF2 | G3-AF2-I16 | G3 | OMU male |
| G3 | AF3 | G3-AF3-I16 | G3 | OMU male |
| KO | AF3 | KO-AF3-I16 | SQ | Neighbouring OMU male |
| KO | AF4 | KO-AF4-I16 | BG | AMU male |
| LZ | AF1 | LZ-AF1-I16 | LZ | OMU male |
| LZ | AF2 | LZ-AF2-I16 | ZD | Neighbouring OMU male |
| LZ | AF3 | LZ-AF3-I16 | BX | Neighbouring OMU male |
| LZ | AF4 | LZ-AF4-I16 | LZ | OMU male |
| SQ | AF4 | SQ-AF4-I16 | SQ | OMU male |
| SQ | AF5 | SQ-AF5-I16 | SQ | OMU male |
| ST | AF2 | ST-AF2-I16 | ST | OMU male |
| SX | AF2 | SX-AF2-I16 | SX | OMU male |
| SX | AF3 | SX-AF3-I16 | SX | OMU male |
| TB | AF1 | TB-AF1-I16 | CM | AMU male |
| TB | AF2 | TB-AF2-I16 | TB | OMU male |
| WF | AF1 | WF-AF1-I16 | WF | OMU male |
| WF | AF2 | WF-AF2-I16 | WF | OMU male |
| XJ | AF1 | XJ-AF1-I16 | LD | Neighbouring OMU male |
| ZB | AF1 | ZB-AF1-I16 | ZB | OMU male |
| ZB | AF2 | ZB-AF2-I16 | XB | AMU male |
| ZD | AF1 | ZD-AF1-I16 | XJ | Neighbouring OMU male |
| ZD | AF2 | ZD-AF2-I16 | ZD | OMU male |
| G3 | AF1 | G3-AF1-I17 | G3 | OMU male |
| HQ | AF1 | HQ-AF1-I17 | EH | AMU male |
| HQ | AF2 | HQ-AF2-I17 | LZ | Neighbouring OMU male |
| ST | AF1 | ST-AF1-I17 | ST | OMU male |
| SX | AF4 | SX-AF4-I17 | SX | OMU male |
| SX | AF5 | SX-AF5-I17 | TB | Neighbouring OMU male |
| TB | AF3 | TB-AF3-I17 | TB | OMU male |
| TB | AF4 | TB-AF4-I17 | TB | OMU male |
| WF | AF3 | WF-AF3-I17 | WF | OMU male |
| XJ | AF2 | XJ-AF2-I17 | XJ | OMU male |
| XJ | AF3 | XJ-AF3-I17 | XJ | OMU male |
| ZD | AF3 | ZD-AF3-I17 | XJ | Neighbouring OMU male |
| ZD | AF4 | ZD-AF4-I17 | WX | AMU male |
| ZD | AF5 | ZD-AF5-I17 | ZD | OMU male |

**Supplementary Table 2.** Association between MHC-heterozygosity and the probability of one male being an OMU male of an EPO or a genetic father of an EPO, given random effects of female ID, male ID and sampling years.

|  | Estimate | Std. Error | t | *P* |
| --- | --- | --- | --- | --- |
| (Intercept) | 0.669 | 0.297 | 2.255 | 0.045 |
| *H*_MHC_-*DQA1* | -0.068 | 0.441 | -0.154 | 0.880 |
| *H*_MHC_-*DQB1* | -0.156 | 0.344 | -0.454 | 0.655 |

**Supplementary Table 3.** Association between the evolutionary amino acid distance of individual MHC alleles (intra-*E*_aadis_) and the probability of one male being an OMU male of an EPO or a genetic father of an EPO, given random effects of female ID, male ID and sampling years.

|  | Estimate | Std. Error | t | *P* |
| --- | --- | --- | --- | --- |
| (Intercept) | 0.666 | 0.185 | 3.599 | 0.003 |
| intra-*E*_aadis_-*DQA1* | 0.245 | 1.601 | 0.153 | 0.880 |
| intra-*E*_aadis_-*DQB1* | -1.534 | 1.662 | -0.923 | 0.368 |

**Supplementary Table 4.** Association between the functional amino acid distance of individual MHC alleles (intra-*F*_aadis_) and the probability of one male being an OMU male of an EPO or a genetic father of an EPO, given random effects of female ID, male ID and sampling years.

|  | Estimate | Std. Error | t | *P* |
| --- | --- | --- | --- | --- |
| (Intercept) | 0.678 | 0.183 | 3.700 | 0.003 |
| intra-*F*_aadis_-*DQA1* | 0.000 | 0.005 | 0.105 | 0.917 |
| intra-*F*_aadis_-*DQB1* | -0.004 | 0.004 | -0.892 | 0.383 |

**Supplementary Table 5.** Association between the MHC genetic similarity of females and males (*D*_FM_) and the probability of one male being an OMU male of an EPO or a genetic father of an EPO, given random effects of female ID, male ID and sampling years.

|  | Estimate | Std. Error | t | *P* |
| --- | --- | --- | --- | --- |
| (Intercept) | 0.523 | 0.138 | 3.795 | 0.001 |
| *D*_FM_-*DQA1* | 0.431 | 0.345 | 1.249 | 0.219 |
| *D*_FM_-*DQB1* | -0.473 | 0.277 | -1.706 | 0.096 |

**Supplementary Table 6.** Association between pairwise MHC evolutionary amino acid distance (*E*_aadis_) and the probability of one male being an OMU male of an EPO or a genetic father of an EPO, given random effects of female ID, male ID and sampling years.

|  | Estimate | Std. Error | t | *P* |
| --- | --- | --- | --- | --- |
| (Intercept) | 0.431 | 0.231 | 1.864 | 0.072 |
| *E*_aadis_-*DQA1* | -0.829 | 0.758 | -1.093 | 0.282 |
| *E*_aadis_-*DQB1* | 0.980 | 0.770 | 1.272 | 0.211 |

**Supplementary Table 7.** Association between pairwise MHC functional amino acid distance (*F*_aadis_) and the probability of one male being an OMU male of an EPO or a genetic father of an EPO, given random effects of female ID, male ID and sampling years.

|  | Estimate | Std. Error | t | *P* |
| --- | --- | --- | --- | --- |
| (Intercept) | 0.427 | 0.228 | 1.875 | 0.071 |
| *F*_aadis_-*DQA1* | -0.002 | 0.002 | -1.112 | 0.274 |
| *F*_aadis_-*DQB1* | 0.003 | 0.002 | 1.285 | 0.207 |

**Supplementary Table 8.** Association between overall heterozygosity and the probability of one male being an OMU male of an EPO or a genetic father of an EPO, given random effects of female ID, male ID and sampling years.

|  | Estimate | Std. Error | t | *P* |
| --- | --- | --- | --- | --- |
| (Intercept) | 0.111 | 0.591 | 0.187 | 0.854 |
| *MLH* | 0.631 | 0.984 | 0.641 | 0.529 |

**Supplementary Table 9.** Association between genetic relatedness and the probability of one male being an OMU male of an EPO or a genetic father of an EPO, given random effects of female ID, male ID and sampling years.

|  | Estimate | Std. Error | t | *P* |
| --- | --- | --- | --- | --- |
| (Intercept) | 0.479 | 0.099 | 4.825 | 0.000 |
| *r* | 0.181 | 0.287 | 0.631 | 0.532 |

**Supplementary Table 10.** Association between MHC-heterozygosity and the probability of extra-pair copulation, given random effects of female ID, male ID and sampling years.

|  | Estimate | Std. Error | t | *P* |
| --- | --- | --- | --- | --- |
| (Intercept) | 0.140 | 0.227 | 0.615 | 0.547 |
| *H*_MHC_-*DQA1* | 0.321 | 0.379 | 0.845 | 0.407 |
| *H*_MHC_-*DQB1* | -0.005 | 0.329 | -0.014 | 0.989 |

**Supplementary Table 11.** Association between the evolutionary amino acid distance of individual MHC alleles (intra-*E*_aadis_) and the probability of extra-pair copulation, given random effects of female ID, male ID and sampling years.

|  | Estimate | Std. Error | t | *P* |
| --- | --- | --- | --- | --- |
| (Intercept) | 0.495 | 0.210 | 2.357 | 0.027 |
| intra-*E*_aadis_-*DQA1* | -1.331 | 1.814 | -0.734 | 0.574 |
| intra-*E*_aadis_-*DQB1* | 0.913 | 1.853 | 0.493 | 0.711 |

**Supplementary Table 12.** Association between the functional amino acid distance of individual MHC alleles (intra-*F*_aadis_) and the probability of extra-pair copulation, given random effects of female ID, male ID and sampling years.

|  | Estimate | Std. Error | t | *P* |
| --- | --- | --- | --- | --- |
| (Intercept) | 0.482 | 0.195 | 2.473 | 0.161 |
| intra-*F*_aadis_-*DQA1* | -0.003 | 0.005 | -0.68 | 0.581 |
| intra-*F*_aadis_-*DQB1* | 0.002 | 0.005 | 0.449 | 0.707 |

**Supplementary Table 13.** Association between the MHC genetic similarity of females and males (*D*_FM_) and the probability of extra-pair copulation, given random effects of female ID, male ID and sampling years.

|  | Estimate | Std. Error | t | *P* |
| --- | --- | --- | --- | --- |
| (Intercept) | 0.403 | 0.120 | 3.364 | 0.004 |
| *D*_FM_-*DQA1* | 0.262 | 0.314 | 0.834 | 0.409 |
| *D*_FM_-*DQB1* | -0.262 | 0.314 | -0.834 | 0.409 |

**Supplementary Table 14.** Association between pairwise MHC evolutionary amino acid distance (*E*_aadis_) and the probability of extra-pair copulation, given random effects of female ID, male ID and sampling years.

|  | Estimate | Std. Error | t | *P* |
| --- | --- | --- | --- | --- |
| (Intercept) | 0.401 | 0.12 | 3.345 | 0.003 |
| *E*_aadis_-*DQA1* | 0 | 0.002 | -0.004 | 0.997 |
| *E*_aadis_-*DQB1* | 0 | 0.002 | 0.004 | 0.997 |

**Supplementary Table 15.** Association between pairwise MHC functional amino acid distance (*F*_aadis_) and the probability of extra-pair copulation, given random effects of female ID, male ID and sampling years.

|  | Estimate | Std. Error | t | *P* |
| --- | --- | --- | --- | --- |
| (Intercept) | 0.427 | 0.142 | 3.005 | 0.887 |
| *F*_aadis_-*DQA1* | 0 | 0 | 0 | 1 |
| *F*_aadis_-*DQB1* | 0 | 0 | 0 | 1 |

**Supplementary Table 16.** Association between overall heterozygosity and the probability of extra-pair copulation, given random effects of female ID, male ID and sampling years.

|  | Estimate | Std. Error | t | *P* |
| --- | --- | --- | --- | --- |
| (Intercept) | 0.027 | 0.837 | 0.032 | 0.998 |
| *MLH* | 0.622 | 1.386 | 0.449 | 0.988 |

**Supplementary Table 17.** Association between genetic relatedness and the probability of extra-pair copulation, given random effects of female ID, male ID and sampling years.

|  | Estimate | Std. Error | t | *P* |
| --- | --- | --- | --- | --- |
| (Intercept) | 0.415 | 0.105 | 3.937 | 0.003 |
| *r* | 0.350 | 0.244 | 1.435 | 0.159 |
